# IL-22 promotes the progression of breast cancer through regulating HOXB-AS5

**DOI:** 10.18632/oncotarget.22063

**Published:** 2017-10-19

**Authors:** Jiang Rui, Zhao Chunming, Gao Binbin, Shao Na, Wang Shengxi, Song Wei

**Affiliations:** ^1^ Department of Oncology, Shandong Provincial Hospital Affiliated to Shandong University, Jinan, 250021, China; ^2^ Department of Opthalmology, Shandong Provincial Hospital Affiliated to Shandong University, Jinan, 250021, China

**Keywords:** IL-22, HOXB-AS5, breast cancer, PI3K/AKT/mTOR

## Abstract

Interleukin-22 (IL-22) is a well-known tumor related inflammatory factor that is associated with variety of cancers. HOXB-AS5, a long non-coding RNA located in HOX gene clusters, has been elevated in breast cancer (BC) tissues. Herein, IL-22 and HOXB-AS5 were upregulated in the serum and tissues of BC patients and were associated with clinical stages. Furthermore, we also investigated the effects of IL-22-HOXB-AS5 pathway on progression of BC, and the results suggested that IL-22 and HOXB-AS5 synergistically promoted MDA-MB-231 cell growth, migration and invasion and activated the PI3K-AKT-mTOR pathway. These findings demonstrated that the IL-22-HOXB-AS5-PI3K/AKT functional axes may serve as potential molecule biomarkers for diagnosis and therapy evaluation or targeted therapeutic strategy in BC.

## INTRODUCTION

Breast cancer (BC) is the most common cancer and is the principal cause of cancer death among females worldwide [1]. Currently, compared to the traditional pathological classification, molecular analysis has a considerable influence on the current understanding of BC biological characteristics [2]. To date, there are four main BC subtypes, defined as luminal A, luminal B, HER-2 enriched and basal-like [3]. Moreover, BC is a heterogeneous disease with numerous gene variations, potential providing clinically relevant information and targeted therapy [4].

Interleukin-22 (IL-22), formally referred to as IL-10-related T cell-derived inducible factor (IL-TIF), belongs to the IL-10 family and is released from immune cells, including several types of CD4+ and CD8+ T lymphocytes, γδ T lymphocytes, natural killer T (NKT) cells and group 3 ILCs [5, 6]. IL-22 binds to the heterodimer formed by the IL-10 receptor b (IL-10Rb) and the IL-22 receptor (IL-22R) to induce differentiation [5, 6]. IL-10Rb is widely expressed on the human cell surface, while IL-22R expression is limited to epithelial cells, but not immune cells [5, 6]. Studies have also shown that IL-22 could modulate the expression of many encoding genes associated with epidermal immunity and remodeling in inflammatory skin diseases [7]. Moreover, the overexpression of IL-22 or its receptor was correlated with tumor progression in digestive cancers, such as pancreatic, gastric and colorectal cancer [8–10]. In addition, Karam et al. recently demonstrated that the IL-22-IL-22R1 pathway could activate extracellular signal-regulated kinase (ERK), c-Jun N-terminal kinase (JNK), and STAT3 signaling pathways through increasing mitogen-activated protein kinase 8 (MAP3K8) phosphorylation to promote epithelial cell transformation, initiation and progression in BC [11].

Long non-coding RNAs (lncRNAs) are longer than 200 nucleotides, with diverse cellular maintenance functions, and the mutation or abnormal expression of these molecules has been associated with the occurrence and development of cancer [12]. The homeobox (HOX) gene family are present on every human chromosome and commonly perform critical functions in embryonic development, thereby affecting the formation of various body structures [13], and the aberrant expression of these genes has been implicated in diverse human diseases, including cancer [3, 14]. A recent report suggested that several non-coding RNAs are located in and associated with HOX gene clusters, including HOXB-AS5 [3]. Another study also reported that HOXB-AS5 was 3.9-fold upregulated in BC tissues compared with matched normal breast tissues [15]. We thereby considered whether the overexpression of HOXB-AS5 gene was associated with BC initiation and progression.

The phosphatidylinositol-3-kinase (PI3K)/AKT signaling pathway plays a central role in the regulation of diverse cellular functions, including proliferation, growth, survival, and metabolism [16]. Genetic aberrations of the PI3K/AKT pathway are among the most commonly observed in human cancer [16]. Mammalian target of the rapamycin (mTOR), a serine/threonine protein kinase, is a downstream effector of AKT, which plays a central role in regulation of cell growth and proliferation [17]. A previous study confirmed that the mechanism of IL-22 regulates BC cell progression through the PI3K/AKT/mTOR pathway. In the present study, we emphatically investigate the functional relationship between IL-22 and HOXB-AS5 in BC initiation and progression.

## RESULT

### IL-22 was upregulated in the serum and tissue of BC patients

**Figure 1:**
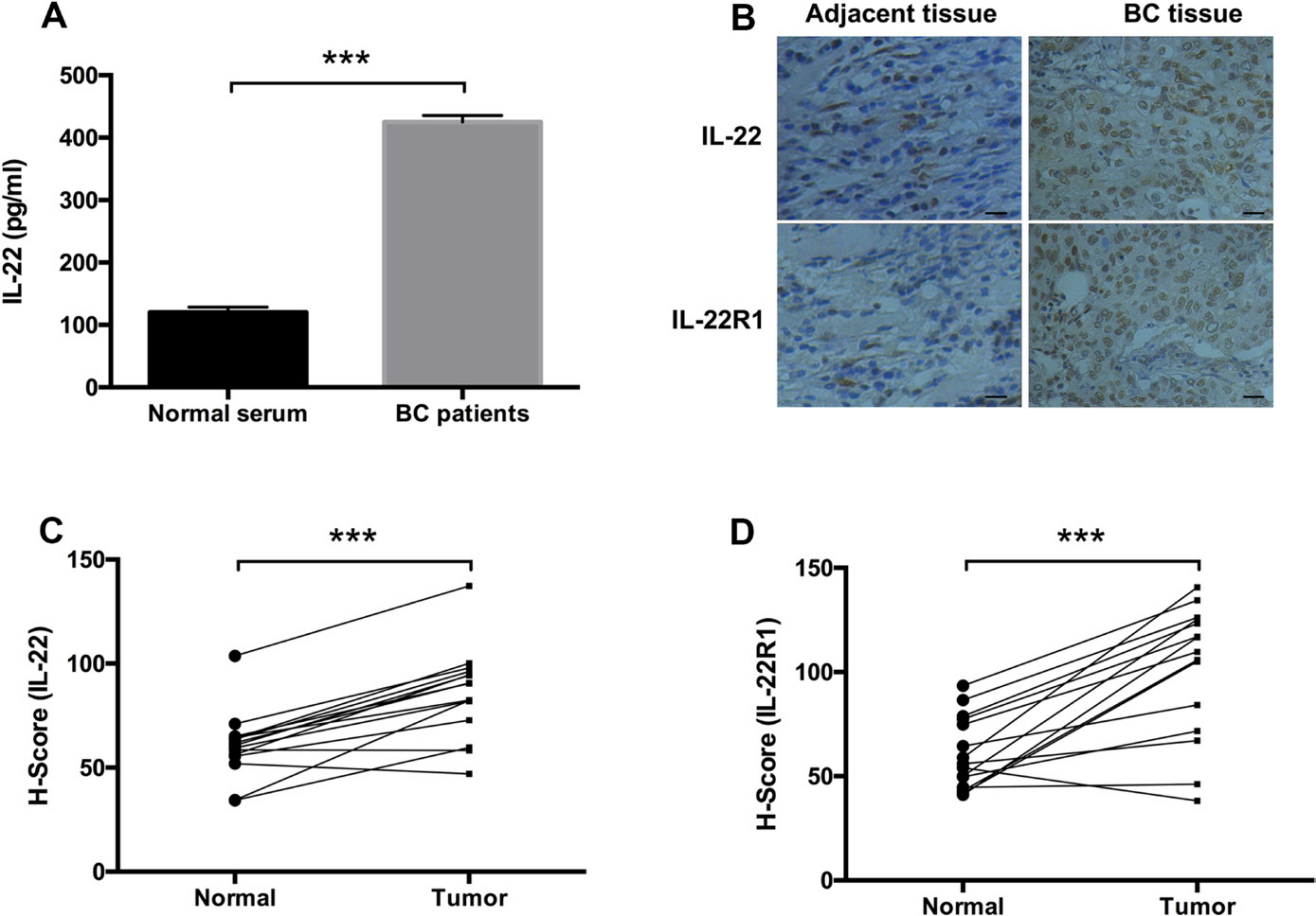
IL-22 was upregulated in the serum and tissue of BC patients (**A**) The concentration of IL-22 in the serum of BC patients and healthy controls were determined by ELISA assay. (**B**) IL-22 and IL-22R1 expression status in BC samples and matched adjacent normal tissues (*n* =15) were detected by IHC assay (Magnification, ×200, Scale bars, 10 µm). (**C**, **D**) Quantified expression levels of IL-22 and IL-22R1 in BC tissues (*n* =15) and corresponding normal breast tissues (*n* =15) detected by IHC assay. Kaplan-Meier survival analysis according to IL-22 and IL-22R1 expression in patients with BC (log- rank test, ***P < 0.001).

To confirm the role of the IL-22-IL-22R1 pathway in BC progression, we detected the expression levels of IL-22 in the serum and tissues of BC patients. The concentration of IL-22 in the serum of BC patients was higher than that in the healthy controls (*P* < 0.001, Figure 1A). Furthermore, IHC assay showed that the expression level of IL-22 was also upregulated in BC tissues compared with matched adjacent normal tissues (*P* < 0.001, Figure 1B, 1C). In addition, IL-22 receptor, IL-22R1, was overexpressed in BC tissue samples (*P* < 0.001, Figure 1B, 1D). These results suggest that the IL-22-IL-22R1 pathway may influence breast tumorigenesis.

### IL-22 could increase the expression of HOXB-AS5 in BC cell lines

**Figure 2:**
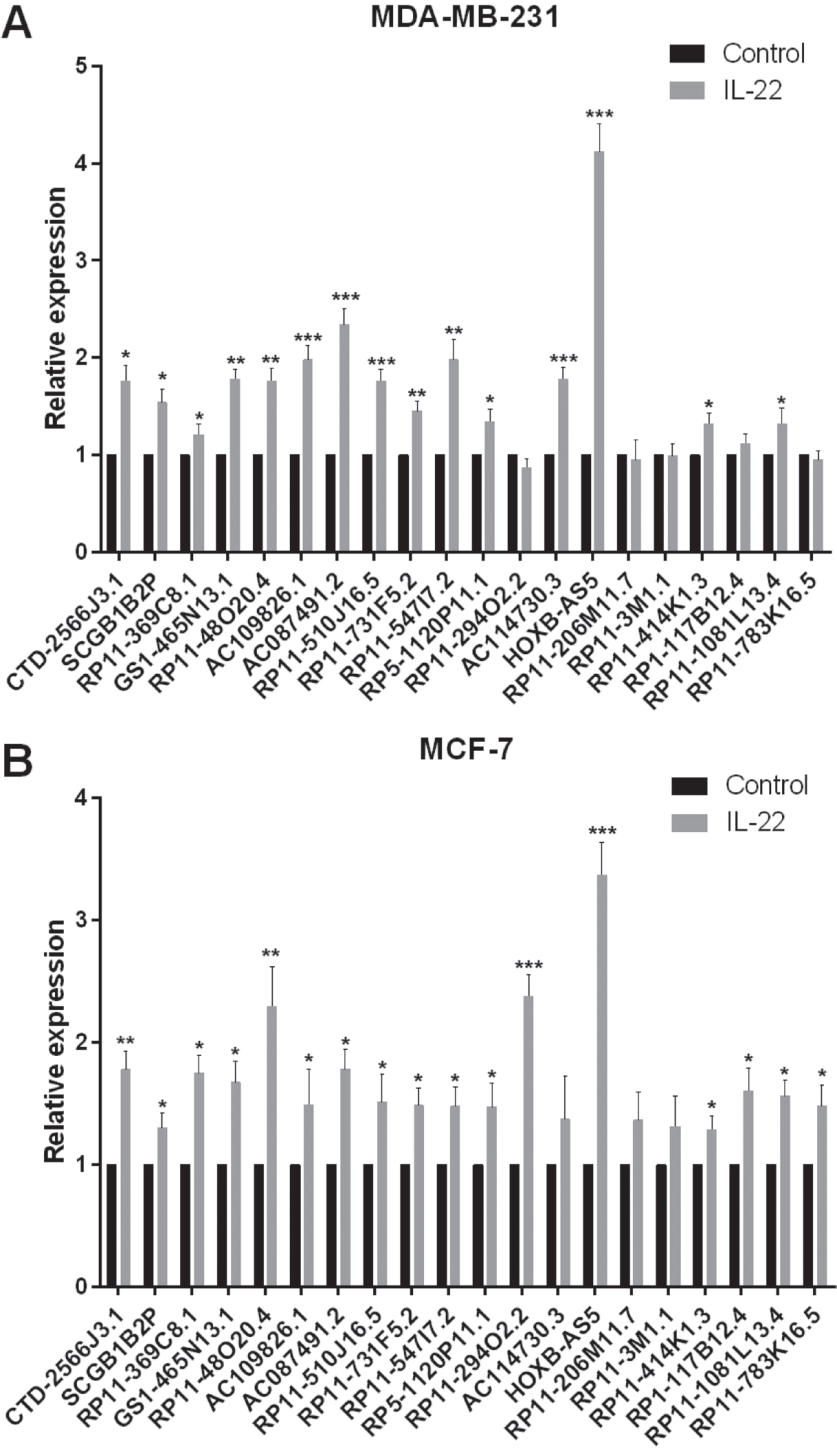
Screen the IL-22 regulated lncRNAs in BC (**A**) Real-time PCR was used to detect the expression of 20 lncRNAs that upregulated in BC tissues by IL-22 treatment in MDA-MB-231 cells (*P < 0.05, **P < 0.01, ***P < 0.001). HOXB-AS5 has a 4.12-fold changes at 24 h after IL-22 treatment compared to control group. (**B**) Real-time PCR was used to detect the expression of 20 lncRNAs that upregulated in BC tissues by IL-22 treatment in MCF-7 cells (* *P* < 0.05, ** *P* < 0.01, *** *P* < 0.001). HOXB-AS5 has a 3.37-fold changes at 24 h after IL-22 treatment compared to control group.

Altered lncRNA expression has been associated with the development of cancer through many signaling pathways. To screen for lncRNAs that may potentially participate in the IL-22-IL-22R1 pathway during breast cancer progression, we treated MDA-MB-231 and MCF-7 cells with PBS (control) or IL-22, and detected expression changes of the top 20 upregulated lncRNAs in BC [15]. As shown in Figure 2A and 2B, HOXB-AS5 showed the largest increase among these lncRNAs (***P < 0.001). Thus, we selected HOXB-AS5 for further study.

### HOXB-AS5 was upregulated in the serum and tissue of BC patients and involved in poor prognosis

**Figure 3:**
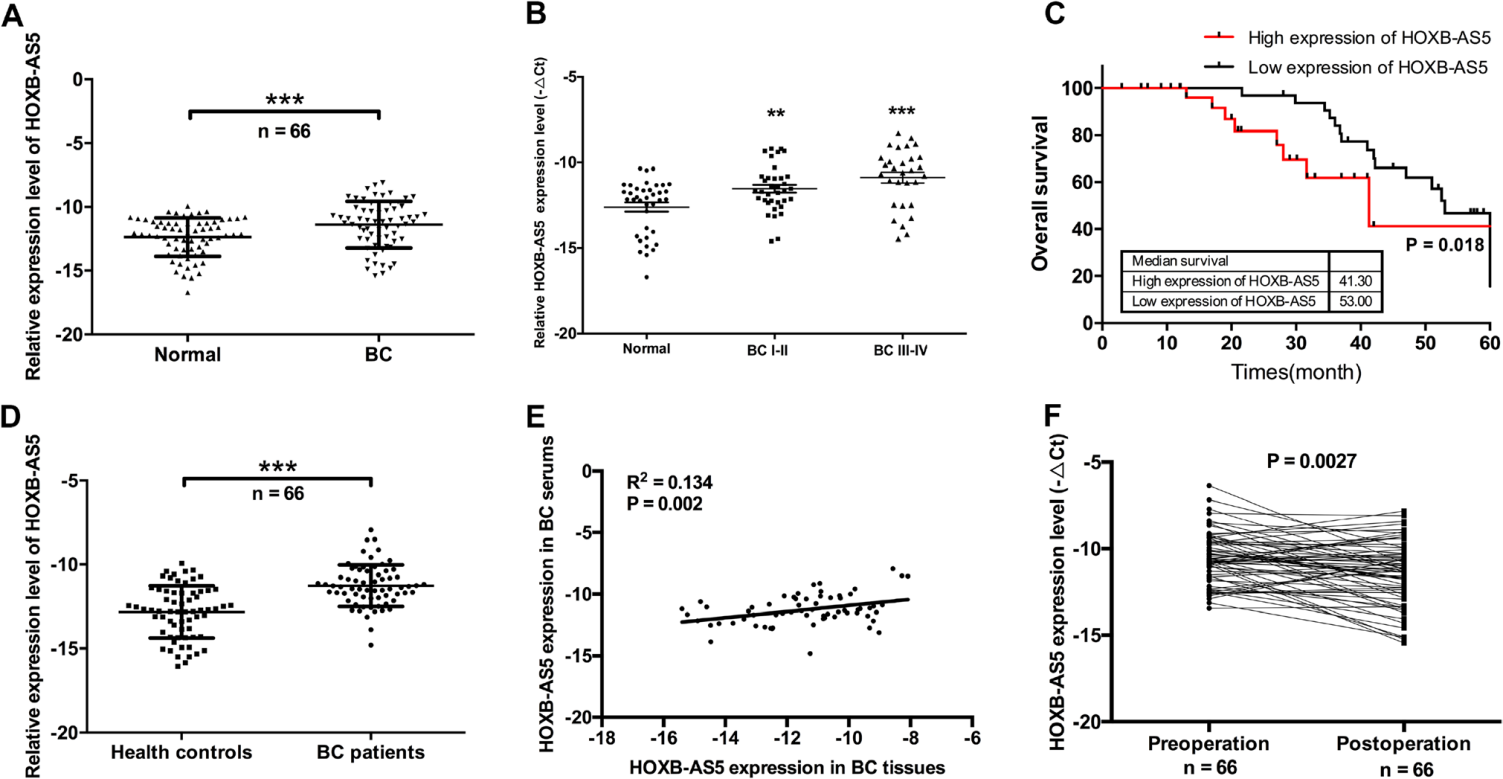
HOXB-AS5 was upregulated in the serum and tissue of BC patients and involved in poor prognosis (**A**) Real-time PCR was used to assay the expression levels of HOXB-AS5 in BC samples and corresponding normal breast tissues (*n* = 66). (**B**) HOXB-AS5 expression levels were detected by real-time PCR in tissue samples from BC patients (*n* = 66) with different stages and normal breast tissues (*n* = 38). (**C**) The 66 cases of BC samples were categorized into high expression group (*n* = 33) and low expression group (*n* = 33) for HOXB-AS5. Kaplan-Meier survival analysis was performed according to HOXB-AS5 expression levels in BC patients (log-rank test, *P* = 0.018). (**D**) HOXB-AS5 expression levels in the serum of BC patients (*n* = 66) and health controls (*n* = 66) were assayed by real-time PCR. (**E**) Correlation analysis of HOXB-AS5 expression between BC tissue and the serum of BC patients (R^2^ = 0.134, *P* = 0.002). (**F**) Real-time PCR was used to assay the variation in the expression levels of HOXB-AS5 between pre-operation and post-operation in the serum of BC patients (*n* = 66).

To investigate the correlation between HOXB-AS5 level and BC progression, we used real-time PCR to examine the expression of HOXB-AS5 in 66 cases of BC samples and corresponding non-tumor tissues. HOXB-AS5 exhibited a higher expression level in BC tissues than in the non-tumor tissues (*P* < 0.001, Figure 3A), and the expression level of HOXB-AS5 was corrected with tumor stages (*P* < 0.001, Figure 3B). Furthermore, Kaplan-Meier analysis indicated the mean survival time for BC patients with high expression of HOXB-AS5 was 41.3 months compared with 53 months for BC patients with low HOXB-AS5 expression (*P* = 0.018, log-rank test, Figure 3C). Next, we detected HOXB-AS5 expression levels in the serum of BC patients. Similarly, HOXB-AS5 was upregulated in the serum of BC patients compared with the healthy controls (*P* < 0.001, Figure 3D). Furthermore, a positive correlation in HOXB-AS5 expression was observed between the serum and tissue of BC patients (R^2^ = 0.134, *P* = 0.002, Figure 3E). To further explore the role of HOXB-AS5 in BC progression, we detected the expression levels of HOXB-AS5 in the serum of pre-operation or post-operation BC patients, and the results showed that surgical treatment could decrease HOXB-AS5 expression in the serum of BC patients (*P* = 0.0027, Figure 3F). These results suggest that HOXB-AS5 is upregulated in both the serum and tissue of BC patients, and higher expression of HOXB-AS5 is associated with shorter patient survival.

### IL-22 promoted cell proliferation of BC by regulated HOXB-AS5

**Figure 4:**
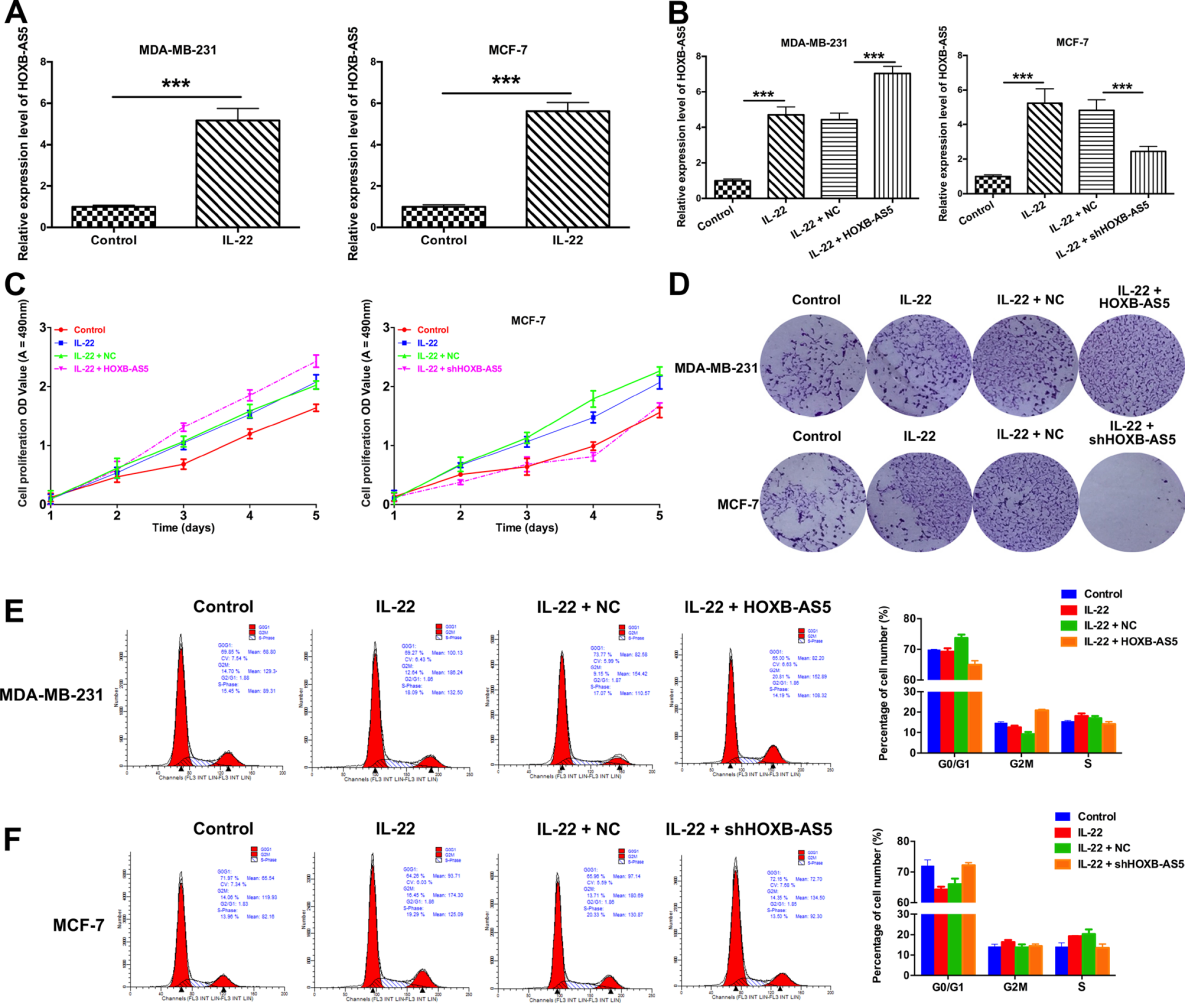
IL-22 increased HOXB-AS5 expression and promoted proliferation and cell cycle entry in BC cells MDA-MB-231 were treated with PBS (control), IL-22, IL-22 and negative control (NC), IL-22 and HOXB-AS5 lentivirus respectively; MCF-7 cells were treated with PBS (control), IL-22, IL-22 and negative control (NC), IL-22 and shHOXB-AS5 lentivirus respectively. (**A**) Real-time PCR was used to detect the expression level of HOXB-AS5 in MDA-MB-231 and MCF-7 cells treated with IL-22 or PBS (control) (*** *P* < 0.001). (**B**) The expression level of HOXB-AS5 was detected by Real-time PCR in MDA-MB-231 and MCF-7 cells (*** *P* < 0.001). (**C**) MTT assay was used to detect the cell proliferation ability in MDA-MB-231 and MCF-7 cells (*** *P* < 0.001). (**D**) The colony formation assay was performed to detect the clonogenic capacity in MDA-MB-231 and MCF-7 cells. (**E**) Cell cycle distribution was analyzed using flow cytometry with propidium iodide staining in MDA-MB-231 and MCF-7 cells (* *P* < 0.05).

First, we treated MDA-MB-231 and MCF-7 cells with PBS (control) or IL-22, and observed that HOXB-AS5 was significantly upregulated in the presence of IL-22 (*P* < 0.001, Figure 4A). We subsequently infected MDA-MB-231 and MCF-7 cells with Lenti-HOXB-AS5 and Lenti-shHOXB-AS5, respectively. As shown in Figure 4B, the expression level of HOXB-AS5 was significantly higher with IL-22 treatment and much higher after transfection with Lenti-HOXB-AS5 in MDA-MB-231 cells (*P* < 0.001, Figure 4B, left). In MCF-7 cells infected with Lenti-shHOXB-AS5, the expression of HOXB-AS5 induced through IL-22 could be reversed via HOXB-AS5 knockdown (*P* < 0.001, Figure 4B, right), indicating that IL-22 could specifically upregulate the expression of HOXB-AS5 in BC cells.

To investigate whether IL-22 and HOXB-AS5 affect BC cell growth, we performed an MTT assay to detect the proliferation of MDA-MB-231 and MCF-7 cells and observed that IL-22 and HOXB-AS5 could synergistically enhance the growth of MDA-MB-231 cells (*P* < 0.001, Figure 4C left), and inhibition of HOXB-AS5 could block this effect in MCF-7 cells (*P* < 0.001, Figure 4C right). The clonogenic potential of cancer cells is correlated with tumor formation *in vivo* [18, 19]. Therefore, we employed a colony formation assay to determine whether IL-22 and HOXB-AS5 affect the clonogenic capacity of BC cells. As shown in Figure 4D, MDA-MB-231 and MCF-7 cells treated with IL-22 exhibited larger focus numbers compared with the control cells. Moreover, the effect of IL-22 was dramatically increased as a result of the overexpression of HOXB-AS5 in MDA-MB-231 cells, causing a sizeable number of colonies compared with MDA-MB-231 cells treated with IL-22 alone (Figure 4D up). Whereas the inhibition of HOXB-AS5 eliminated the enhanced clonogenic capacity induced through IL-22 in MCF-7 cells (Figure 4D down). These results suggest that the expression of HOXB-AS5 can be positively regulated through IL-22 and is required for IL-22-stimulated proliferation and focus formation in BC cells.

### IL-22 promoted cell cycle entry and prevented cellular apoptosis though HOXB-AS5 in BC cells

To understand how IL-22 and HOXB-AS5 control the growth of BC cells, we determined the cell cycle distribution of BC cells using flow cytometry. The results are shown in Figure 4E and 4F. The proportion of the G0/G1 phases in MDA-MB-231 cells treated with PBS (control), IL-22, IL-22 and negative control (NC), IL-22 and Lenti-HOXB-AS5 were 69.85% ± 4.2%, 69.27% ± 4.6%, 73.77% ± 2.3% and 65.0% ± 2.3%, respectively, and 71.97% ± 4.8%, 64.26% ± 3.9%, 65.96% ± 1.8% and 72.16% ± 1.8% in MCF-7 cells treated with PBS (control), IL-22, IL-22 and negative control (NC), IL-22 and shHOXB-AS5 lentivirus, respectively (Figure 4E). Taken together, HOXB-AS5 enhanced IL-22-stimulated cell cycle entry from G0/G1 to S phase in MDA-MB-231 cells, whereas the inhibition of HOXB-AS5 neutralized this effect in MCF-7 cells (*P* < 0.05, Figure 4F).

**Figure 5:**
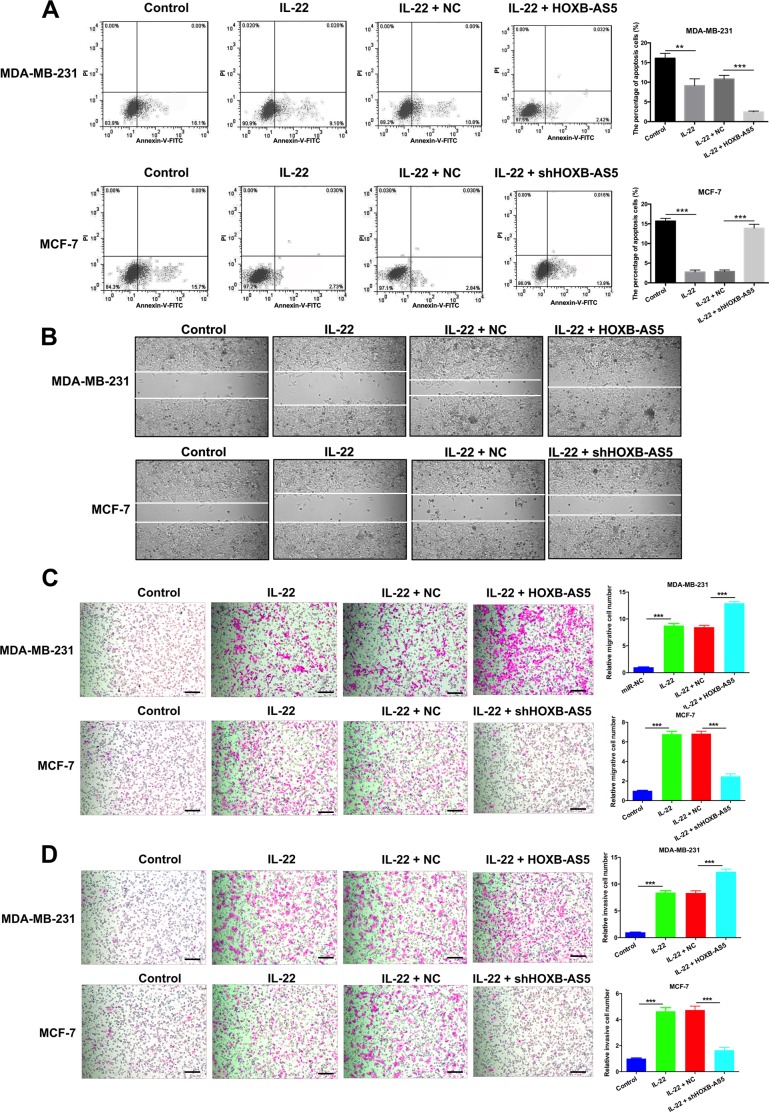
IL-22 prevented cellular apoptosis and promoted migration and invasion though HOXB-AS5 in BC cells MDA-MB-231 were treated with PBS (control), IL-22, IL-22 and negative control (NC), IL-22 and HOXB-AS5 lentivirus respectively; MCF-7 cells were treated with PBS (control), IL-22, IL-22 and negative control (NC), IL-22 and shHOXB-AS5 lentivirus respectively. (**A**) The total apoptosis cells were determined using flow cytometry with PI staining and FACS analysis in MDA-MB-231 and MCF-7 cells. (**B**) Wound healing assay was used to detect the motor ability of MB-231 and MCF-7 cells (Magnification, ×200, Scale bars, 10 µm). (**C**) Transwell assay was used to detect the migration of MB-231 and MCF-7 cells (Magnification, ×200, Scale bars, 10 µm, *** *P* < 0.001). (**D**) Cell invasion ability was detected by Transwell assay in MB-231 and MCF-7 cells (Magnification, ×200, Scale bars, 10 µm, *** *P* < 0.001).

To further explore the potential mechanism responsible for IL-22 and HOXB-AS5-mediated BC cell growth promotion, flow cytometry was performed to evaluate cellular apoptosis with PI staining and FACS analysis in MDA-MB-231 and MCF-7 cells. The proportion of the apoptotic cells in MDA-MB-231 cells treated with PBS (control), IL-22, IL-22 and negative control (NC), IL-22 and Lenti-HOXB-AS5 was 16.1% ± 0.2%, 9.1% ± 0.6%, 10.8% ± 2.3% and 2.42% ± 2.3%, respectively, and whereas 15.7% ± 0.8%, 2.73% ± 0.9%, 2.84% ± 1.8% and 13.9% ± 1.8% in MCF-7 cells treated with PBS (control), IL-22, IL-22 and negative control (NC), IL-22 and shHOXB-AS5 lentivirus, respectively. Fewer apoptotic cells were detected in BC cells treated with IL-22, and overexpressing HOXB-AS5 could dramatically reduce cell death, whereas the inhibition of HOXB-AS5 neutralized this effect (*P* < 0.001 Figure 5A). These findings indicate that the IL-22 and HOXB-AS5 act synergistically to enhance cell cycle entry from G0/G1 to S phase and prevent cellular apoptosis in BC cells.

### IL-22 promoted migration and invasion though HOXB-AS5 in BC cells

Metastasis is one of the major characteristics of malignancy. Therefore, we performed a wound healing assay to detect the motility of MB-231 and MCF-7 cells. Compared with the controls, BC cells treated with IL-22 showed a narrower wound area at 24 hours after cell propagation, and overexpressing HOXB-AS5 enhanced IL-22-stimulated cell motility whereas the inhibition of HOXB-AS5 would reverse this phenomenon (Figure 5B). Since cell migration and invasion abilities have a decisive influence on metastases, we further investigated cell invasiveness using a Transwell assay. MDA-MB-231 and MCF-7 cells treated with IL-22 showed a significant increase in the number of migrated cells compared with the controls (***P < 0.001, Figure 5C). Consistent with the findings of the migration assay, BC cells treated with IL-22 exhibited a significant increase in cell invasion ability compared with the controls (***P < 0.001, Figure 5D). Additionally, as shown in Figure 5C and 5D, overexpressed HOXB-AS5 cooperated with IL-22 to enhance the migration and invasion of MDA-MB-231 cells (***P < 0.001, Figure 5C). In contrast, knockdown of HOXB-AS5 resisted the cell invasiveness induced through IL-22 in MCF-7 cells (***P < 0.001, Figure 5D). Collectively, these results imply that IL-22 promoted migration and invasion, in part, through HOXB-AS5 in BC cells.

### IL-22 activated PI3K-AKT-mTOR pathway though HOXB-AS5 in BC cells

**Figure 6:**
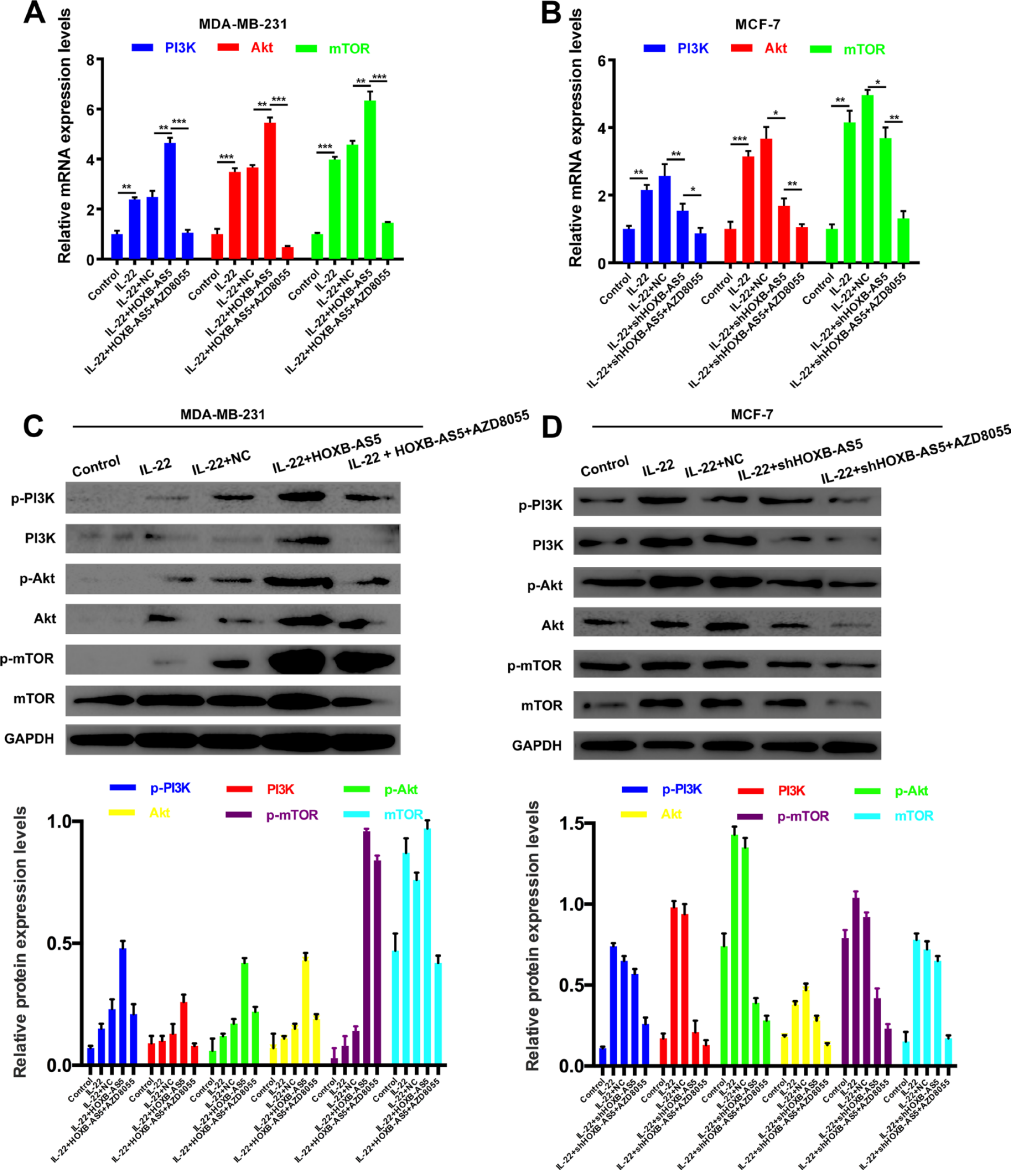
IL-22 activated PI3K-AKT-mTOR pathway though HOXB-AS5 in BC cells MDA-MB-231 cells were treated with PBS (control), IL-22, IL-22 and negative control (NC), IL-22 and Lenti-HOXB-AS5, IL-22 and Lenti-HOXB-AS5 as well as AZD8055, respectively; MCF-7 cells were treated with PBS (control), IL-22, IL-22 and negative control (NC), IL-22 and Lenti-shHOXB-AS5, IL-22 and Lenti-shHOXB-AS5 as well as AZD8055, respectively. (**A**–**B**) qRT-PCR assay was used to analyze the the mRNA expression levels of PI3K, AKT, and mTOR. (**C**–**D**) Western Blot assay was performed to detect the protein expression levels of p-PI3K, PI3K, p-AKT, AKT, p-mTOR and mTOR. Quantitative data were analyzed according to the grey value.

**Figure 7:**
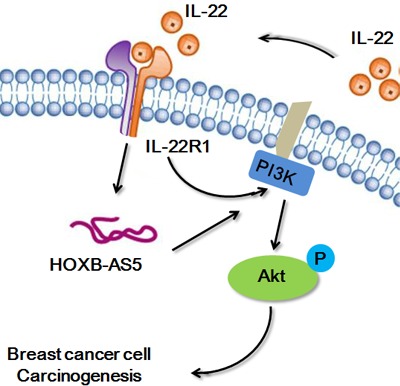
The network model of IL-22/IL22R1/HOXB-AS5 in breast cancer

Mitra et al. showed that IL-22 induced the proliferation of normal human epidermal keratinocytes (NHEK) and human keratinocytes and fibroblast-like synoviocyte (FLS) cells is dependent on the PI3K/Akt/mTOR signaling pathway [20]. Thus, in the present study, we performed real-time PCR (Figure 6A, 6B) and western blot assays (Figure 6C, 6D) to investigate the correlation between IL-22, HOXB-AS5 and the PI3K/Akt/mTOR signaling pathway. As shown in Figure 6, phosphorylated molecules of the PI3K/Akt/mTOR pathway, and p-PI3K, p-AKT and p-mTOR were upregulated in BC cells treated with IL-22, and increased expression was observed in the presence of both IL-22 and HOXB-AS5 overexpression. BC cells transfected with Lenti-shHOXB-AS5 or treated with AZD8055 could inhibit IL-22-stimulated p-PI3K, p-AKT and p-mTOR upregulation. Based on these results, we concluded that HOXB-AS5 could synergistically facilitate the activation of the PI3K/Akt/mTOR pathway caused by IL-22, and the inhibition of HOXB-AS5 or PI3K/mTOR could block this effect. Based on a previous study showing that the PI3K-AKT-mTOR signaling pathway plays a critical role in the moderation of proliferation, survival and metabolism in BC [21], we concluded that IL-22 likely promotes the proliferation and invasion of BC cells in a HOXB-AS5-dependent manner through the activation of the PI3K- AKT-mTOR pathway (Figure 7).

## DISCUSSION

BC is a common neoplastic disease with heterogeneous pathogenesis. Among the inductive factors in the occurrence of tumors, inflammatory mediators and cell effectors are important components of the local environment of tumors [22]. IL-22, responsible for epithelial remodeling and inflammation [23], plays an important role in a variety of autoimmune diseases, malignant tumors and infectious diseases. Elevated IL-22 expression has been detected in several human tumors, including ovarian, prostate, breast, hepatocellular, esophageal, gastric, and non-melanoma skin cancers [5]. In a previous study and in the present study, IL-22 was upregulated in the serum and tissue of BC patients. In addition, IL-22R1 is also overexpressed in BC samples. IL-22 binds to the receptor complex comprising IL-10R2 and IL-22R1 to activate the transcription factor STAT-3 [5, 6] and promote tumor progression in several cancer types, including colorectal cancer and BC [11, 24–26]. Notably, IL-22R1 is uniquely and exclusively expressed on epithelial and tissue cells, but not immune cells, making this receptor an ideal therapeutic target with less side effects to potentially achieve antitumor immunity [27].

Evidence has shown that IL-22 has a crucial effect on non-melanoma skin cancer cell proliferation and the metastasis of colon and lung cancers [27, 28]. Concomitantly, we performed numerous experiments, including cell proliferation, colony formation, cell cycle and apoptosis, cell motility, migration and invasion tests, to assess the effect of IL-22 on the proliferation and invasiveness of BC cell lines MDA-MB-231 and MCF7. As shown in Figures 3 and 4, cell proliferation, colony formation, cell cycle entry, apoptosis inhibition, cell motility, migration and invasion abilities were significantly elevated in BC cells treated with IL-22 compared with the control cells treated with PBS. These data are consistent with a previous report showing that elevated IL-22 expression promotes tumor progression in BC.

HOX genes generally function as transcriptional regulators during normal morphogenesis in cell-to-cell communication processes; the modification of which may lead to the progression of cancer [29, 30]. The HOX gene homology domain binds to specific DNA sequences and regulates gene transcription [31]. However, the potential mechanisms underlying the function of HOX genes in tumorigenesis have not yet been elucidated. LncRNAs have been frequently investigated in recent years and certain lncRNAs associated with HOX genes have been detected in sequencing and ChIP platform research [3]. One of these lncRNAs, HOXB-AS5, is 3.9-fold elevated in BC tissues compared with matched normal breast tissues [15]. Hence, we investigated the clinicopathological significance of HOXB-AS5 expression in BC patients. Interestingly, HOXB-AS5 was overexpressed in the serum and tissues of BC patients, and higher expression was observed in advanced stages than in the lower stages. Moreover, he high expression of HOXB-AS5 is associated with shorter patient survival, and the expression of HOXB-AS5 in the serum was positively correlated with the tissues of BC patients. In addition, surgical treatment could decrease HOXB-AS5 expression in the serum of BC patients. Taken together, these findings implicate HOXB-AS5 as a potential biomarker in BC diagnosis and therapy evaluation.

To date, there are no reports concerning HOXB-AS5 and tumorigenesis, unless it is upregulated in BC tissues. Therefore, we determined the effect of HOXB-AS5 on BC progression through the overexpression and knockdown of the HOXB-AS5 gene using lentivirus vectors. Interestingly, we observed that the upregulation of HOXB-AS5 significantly enhanced the proliferation, colony formation, cell cycle entry, apoptosis inhibition, cell motility, migration and invasion properties of MDA-MB-231 cells, whereas the knockdown of HOXB-AS5 reversed these effects in MCF7 cells.

Importantly, IL-22 positively regulates HOXB-AS5 expression, and these two proteins act synergistically to promote MDA-MB-231 cell progression, while the inhibition of HOXB-AS5 blocks IL-22 stimulated oncogenic effects in MCF7 cells. These results suggest that IL-22 promoted BC cell progression, in part, through HOXB-AS5 in BC cells; however, further evidence is needed to unravel the regulatory mechanism.

The PI3K-AKT-mTOR pathway plays a crucial role in the regulation of critical cellular functions, including survival, proliferation, and metabolism, and the deregulation of this pathway is a common event in neoplastic diseases, including BC [16, 21]. In the present study, we used the ATP-competitive mTOR kinase inhibitory drug AZD8055 [32] to investigate the effects of the IL-22-HOXB-AS5 pathway on the activation of the PI3K-AKT-mTOR pathway. As shown in Figure 5, HOXB-AS5 is required for the IL-22-mediated activation of the PI3K-AKT-mTOR pathway in BC cells. Together with the findings of previous studies, these results suggest that the IL-22-HOXB-AS5-PI3K/AKT functional axis may be one of the carcinogenic mechanisms of BC.

## MATERIALS AND METHODS

### Clinical and histological evaluation of human tissues

The human specimens in the present study were collected from BC patients who received surgery at the Shandong Provincial Hospital Affiliated to Shandong University from Jun 2011 to Jun 2013. None of the patients received preoperative treatment, including chemotherapy or radiotherapy. These 66 BC cases were underwent mastectomy and their clinical characteristics are shown in Table 1. The nontumorous samples were obtained at a distance of at least 5 cm from the tumor, and all tissues were histologically examined.

**Table 1:** The relationship of HOXB-AS5 expression level (dCt) with clinicopathological factors in breast cancer tissues

Characteristics	No. of patients (%)	HOXB-AS5	
		Mean ± SEM	*P* value
**Total no. of patients**	66		
**Age (year)**			
> 60	32 (48.5)	10.21 ± 2.75	0.76
≤ 60	34 (51.5)	11.19 ± 1.75	
**Lymphatic metastasis**			
N0	39 (59.1)	10.45 ± 2.32	0.73
N1–N2	27 (40.9)	9.36 ± 1.74	
**Distal metastasis**			
M0	54 (81.8)	11.58 ± 1.27	0.67
M1	12 (18.2)	10.38 ± 1.37	
**TNM stage**			
0 & I & II	43 (65.2)	9.01 ± 0.89	0.048*
III & IV	23 (34.8)	11.98 ± 1.14	

### Cell culture

Human MDA-MB-231 and MCF-7 cells were obtained from the Type Culture Collection of the Chinese Academy of Sciences (Shanghai, China), and all cells were characterized through mycoplasma detection, DNA-Fingerprinting, isozyme detection and cell vitality detection. MDA-MB-231 and MCF-7 cells were cultured in RPMI 1640 medium (Invitrogen) supplemented with 10% fetal bovine serum (FBS) and 1% penicillin and streptomycin (Thermo Scientific) in cell incubators (Thermo) at 37 °C in a 5% CO_2_ atmosphere. The main method of cell functional study is described in the Supplementary material.

### Lentiviral vector construction and transduction

The HOXB-AS5 sequence was obtained from NCBI. Full-length cDNA was amplified through RT-PCR using the total mRNA of MDA-MB-231 cells. subsequently, the PCR products were inserted into a human U6 promoter-containing pBluescript SK (+) plasmid (pU6). The constructs obtained were cloned into the lentiviral plasmid pLUNIG to achieve the lentiviruses overexpressing HOXB-AS5 (Lenti-HOXB-AS5). Lentiviral vector expressing Enhanced Green Fluorescent Protein (EGFP) was used as the control (NC). Furthermore, we designed a short-hairpin RNA (shRNA) to target human HOXB-AS5, and cloned the shRNA into pU6. Subsequently, the U6-shRNA cassettes were sub-cloned into the lentiviral vector pLUNIG to achieve the lentiviruses carrying shRNA targeting HOXB-AS5 (Lenti-shHOXB-AS5). Lentivirus carrying shRNA targeting firefly luciferase (shNC) was used as the control. The cells were transduced with the lentiviruses using polybrene (8 μg/ml, Sigma-Aldrich, St. Louis, MO).

### Western blot analysis

The cells were lysed on ice in lysis buffer containing protease inhibitor cocktail (Thermo Fisher Scientific, Inc.). The total concentration of protein was detected using the Pierce BCA Protein Assay kit (Thermo Fisher Scientific, Inc.). An equal amount of total protein (50 µg) was separated through SDS-PAGE, and subsequently electrotransferred onto polyvinylidene difluoride membranes (EMD Millipore). After blocking in 5% skimmed milk (BD Biosciences) for 2 hrs. at room temperature and subsequent incubation with primary antibodies overnight at 4°C, the membranes were incubated with the appropriate horseradish peroxidase-conjugated secondary antibody for 1.5 hrs. at room temperature. The protein bands were detected using an ECL system (Amersham Pharmacia Biotech) and quantified using Image Lab Software version 4.1 (BIO RAD).

### Immunohistochemistry(IHC)

Immunohistochemistry was performed on paraformaldehyde-fixed paraffn sections. The TMA blocks were then cut into 4 µM sections for immunostaining. The sections were incubated respectively with IL22 and IL22R1 (ab18498 and ab5984; abcam) antibody overnight at 4°C. Histostain-Plus 3rd Gen IHC Detection Kit (Invitrogen Co., San Diego, CA) was applied for 30 mins to visualize the positive signals.

### Statistical analysis

All experiments were performed at least three times, and numerical data are presented as the means ± standard error of mean (SEM). Statistical analysis was performed using SPSS 13.0 (IBM, Armonk, NY, USA), and statistical significance was assessed using paired two-tailed Student’s *t* test, unpaired two-tailed Student’s t test or analysis of variance. *P* < 0.05 was considered statistically significant.

## CONCLUSIONS

In summary, the results of the present study indicate that IL-22 and HOXB-AS5 are upregulated in both the serum and tissues of BC patients, and the HOXB-AS5 expression can be positively regulated through IL-22 in BC cells. Moreover, the high expression of HOXB-AS5 is associated with clinical stages and shorter patient survival, and the expression of HOXB-AS5 in the serum was positively correlated with the tissues of BC patients. In addition, surgical treatment decreased HOXB-AS5 expression in the serum of BC patients. Furthermore, IL-22 and HOXB-AS5 synergistically promoted MDA-MB-231 cell proliferation, colony formation, cell cycle entry, apoptosis inhibition, cell motility, migration and invasion, and activated the PI3K-AKT-mTOR pathway, while the inhibition of HOXB-AS5 blocked the IL-22-mediated oncogenic effects on MCF7 cells. We therefore conclude that the IL-22-HOXB-AS5-PI3K/AKT functional axis may be one of the carcinogenic mechanisms of BC, serving as potential molecule biomarkers for diagnosis and therapy evaluation or targeted therapeutic strategy in BC.
